# A PROSS-designed extensively mutated estrogen receptor α variant displays enhanced thermal stability while retaining native allosteric regulation and structure

**DOI:** 10.1038/s41598-021-89785-1

**Published:** 2021-05-18

**Authors:** Mark Kriegel, Hanna J. Wiederanders, Sewar Alkhashrom, Jutta Eichler, Yves A. Muller

**Affiliations:** 1 Division of Biotechnology, Department of Biology, Friedrich-Alexander-Universität Erlangen-Nürnberg (FAU), Erlangen, Germany; 2 Department of Chemistry and Pharmacy, Friedrich-Alexander-Universität Erlangen-Nürnberg (FAU), Erlangen, Germany

**Keywords:** Biochemistry, Biotechnology, Structural biology

## Abstract

Protein stability limitations often hamper the exploration of proteins as drug targets. Here, we show that the application of PROSS server algorithms to the ligand-binding domain of human estrogen receptor alpha (hERα) enabled the development of variant ER_PRS*_ that comprises 24 amino acid substitutions and exhibits multiple improved characteristics. The protein displays enhanced production rates in *E. coli*, crystallizes readily and its thermal stability is increased significantly by 23 °C. hERα is a nuclear receptor (NR) family member. In NRs, protein function is allosterically regulated by its interplay with small molecule effectors and the interaction with coregulatory proteins. The in-depth characterization of ER_PRS*_ shows that these cooperative effects are fully preserved despite that 10% of all residues were substituted_._ Crystal structures reveal several salient features, i.e. the introduction of a tyrosine corner in a helix-loop-helix segment and the formation of a novel surface salt bridge network possibly explaining the enhanced thermal stability. ER_PRS*_ shows that prior successes in computational approaches for stabilizing proteins can be extended to proteins with complex allosteric regulatory behaviors as present in NRs. Since NRs including hERα are implicated in multiple diseases, our ER_PRS*_ variant shows significant promise for facilitating the development of novel hERα modulators.

## Introduction

Human estrogen receptor alpha (hERα) belongs to the family of nuclear receptors (NRs). NRs share high sequence and structure homology and function as important gene transcription regulators in metazoans^1^. In homo- and heterodimeric NRs, each protomer displays a similar modular architecture with the most prominent domains being a DNA-binding and a ligand-binding domain (LBD)^1^. The activity of NRs is tightly regulated by their interplay with small molecule effectors and protein binding partners, which regulate the cellular localization and the transcription regulatory activity of NRs^2^. Small molecule effectors acting as either agonists or antagonists bind to an identical pocket in the LBD of NRs. While agonist binding promotes the interaction of the LBD with coregulatory proteins, such as for example the interaction of hERα with the steroid receptor coactivator-2 (SRC-2) protein, binding of antagonists leads to a rearrangement of so-called helix 12 (H12), and this rearrangement precludes any further interaction with coregulators (Supplementary Fig. S1)^2–4^. These structural rearrangements have been shown in detail for hERα but details may differ in other human nuclear receptors^5^. Overall, the function of the LBD is to act as a ligand-triggered protein–protein interaction switch that can be tripped on by agonists and tripped off by antagonists^2,4^.

The human genome encodes for up to 75 different NRs, and NRs are prime drug target proteins because of their manifold involvement in development, cell homeostasis and diseases^6–8^. A textbook success story is the highly efficient regulation of the progesterone receptor by contraceptives^9^. hERα represents an important target on its own since hERα plays a crucial role in breast cancer and osteoporosis in postmenopausal women^10^. Moreover, the discovery of the beneficial effects of tamoxifen in cancer therapy in 1971 initiated an ongoing search for novel and more advanced hERα modulators^11–13^. At the same time, a number of NRs exists, the so-called orphan receptors, for which the cognate ligands remain to be identified^14^. The exploration of NRs as drug targets requires manifold in vitro experiments such as binding and structural studies. However, a prerequisite for such experiments, namely the availability of high amounts of pure proteins, is often hampered by low protein production yields and protein stability issues. Thus, an efficient procedure to design NR variants that show unaltered activity profile but that can be easily produced and robustly handled is very welcome.

Most proteins are only marginally stable^15,16^. Their low overall thermodynamic stability has been attributed to the absence of any evolutionary pressure to select for more stable variants and to the need for proteins to retain conformational flexibility for correct function^17^. One option to overcome the problem of marginal protein stability is to redesign protein sequences using computational methods such as those implemented in the PROSS server^18^. PROSS combines phylogenetic and atomistic approaches for the design of proteins with increased stability. In an initial step, a sequence blast is performed to gather phylogenetic information from homologous protein sequences in order to identify potential amino acid (AA) substitutions that can be expected to not disrupt protein fold and function. Subsequently, a position specific substitution matrix (PSSM) is calculated with these phylogenetic data, and substitutions with a PSSM score > 0 are compared to the native AAs in Rosetta^19,20^. All substitutions with a ΔΔG_calc_ better than − 0.45 of Rosetta energy units are retained, and a final Rosetta combinatorial sequence design is performed with different ΔΔG_calc_ cutoffs and a phylogeny-biased energy function. Overall, this procedure allows for substitutions to be included in the final design that are predicted to be neutral or singly negative according to the Rosetta calculations and are favored by phylogeny^17,18^.

A number of examples have been reported that illustrate the successful application of the PROSS algorithm for the design of stabilized proteins. Among these are a human acetylcholinesterase variant displaying significantly improved production yields in *Escherichia coli* as well as improved versions of a bacterial phosphotriesterase and a human histone deacetylase^18^. More recent examples include the production of a stabilized version of the kinase domain of the tyrosine kinase FLT3 in *E. coli*, as well as stabilized variants of the interleukin hormone IL-24, of the chromosome region maintenance 1 protein (CRM1), of an acetyl-CoA synthetase, of the malaria invasion protein RH5 and of the myocilin olfactomedin domain^21–26^. Furthermore, the PROSS algorithm has been integrated into a computational flow scheme that allowed for the design of two novel hydrolases with TIM-barrel folds^27^.

Here, we applied the PROSS algorithm to generate a significantly more stable variant of the LBD of hERα termed ER_PRS*_. We show that ER_PRS*_ yields higher production rates in *E. coli* and displays a significant increase in thermal stability of ~ 23 °C. At the same time, all structural and functional features of hERα-LBD are retained in ER_PRS*_ as shown by three crystal structure determinations and by an in detail characterization of the effector-binding properties of ER_PRS*_ and the allosteric modulation of coactivator binding by different effectors. Our results demonstrate that the PROSS algorithm can be beneficially applied to a protein that comprises an elaborated allosteric regulation mechanism without affecting any of its functions.

## Results

### PROSS server predictions and bioinformatic assessment

**Figure 1:**
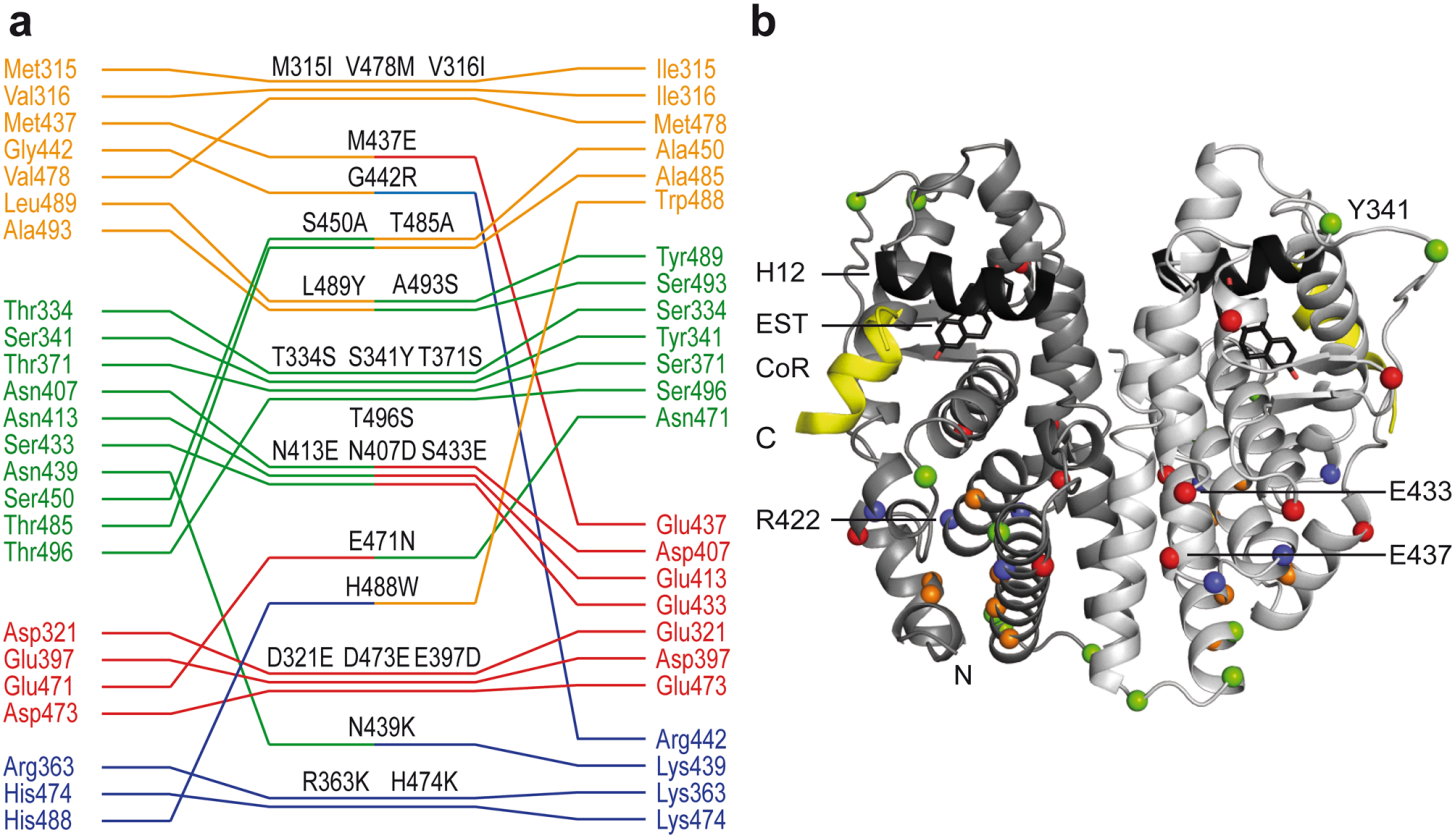
Structural mapping of the PROSS-suggested amino acid substitutions. (**a**) Amino acid substitutions suggested by PROSS and grouped according to the physico-chemical properties of their side chains (hydrophobic: orange, polar: green, acidic: red, basic: blue). (**b**) The hERα-LBD homodimer (in light and dark gray) is shown in the canonical agonist-bound active conformation with helix 12 (H12) and coregulatory protein (CoR) binding highlighted in black and yellow, respectively. The agonist estradiol (EST) is depicted as a stick model. The positions of the substituted amino acids are marked with spheres using the same color code as in panel (**a**).

The PROSS server was used to design a more stable variant of the hERα-LBD for high yield protein production in *E. coli* and for further engineering^25^*.* The PROSS algorithm suggested 24 AA replacements and thereby proposed to substitute as many as 10% of all AAs present in the hERα-LBD (Fig. 1). When classifying these substitutions according to the general chemico-physical properties of AA side chains, i.e. charge, polarity and hydrophobicity, it becomes apparent that the PROSS suggestions cover all possible combinations of class-switching substitutions except for a pure charge reversal (Fig. 1a). Among the most notable exchanges are a replacement of a hydrophobic AA by a negatively charged AA (M437E) and of a backbone flexibility-enhanced glycine by a positively charged AA (G442R). As a net result, the number of charged AAs is increased by four, the number of hydrophobic AAs reduced by one and the number of uncharged polar AAs is reduced by three (Fig. 1a).

No substitutions were allowed near the ligand-binding site, the coregulator-binding site and the dimerization interface in order to preclude changes in the functional behavior of hERα. When taking this into account, it appears that the substitutions are evenly distributed across the entire hERα-LBD (Fig. 1b). A possible trend seems to be that the PROSS algorithm prefers solvent exposed residues since 83% of the substituted AAs are located at the protein’s surface (Fig. 1b). However, if one considers that 74% of the hERα-LBD AAs are classified as non-core residues according to the EPPIC server then this observation seems less significant^28^.

In a first step, the PROSS algorithm performs an automated phylogeny search and clustering analysis to identify potentially beneficial substitutions. This step is followed by partly phylogeny-biased atomistic calculations. To better understand the decision making process of the algorithm and the underlying phylogenetic analysis, all PROSS-suggested substitutions were retrospectively reevaluated with a knowledge-based phylogenetic analysis using the software R^29^. For this purpose, 475 reviewed AA sequences anotated as containing a NR-LBD on PROSITE (PROSITE entry: PS51843) were retrieved and truncated to the respective NR-LBD segment^30^. Duplicates were excluded, and the resulting 422 sequences aligned with ClustalW^31^. With regard to this multiple alignment, the mean relative frequency of all substitutions proposed by the PROSS algorithm is nearly 19%. By contrast, the mean relative frequency of the native AAs initially present at these positions is only 11%. For 46% of all proposed substitutions, the most abundant AA was chosen, and for 75% of the cases, one of the three most frequently observed AAs at a given position was selected (Supplementary Fig. S2). Only one outlier can be identified, namely the PROSS-proposed introduction of Tyr341, which exhibits a relative frequency of only 0.3% at this position in the multiple sequence alignment.

### ER_PRS*_ is properly folded and displays improved thermal stability

Four different protein variants were produced recombinantly in *E. coli* to experimentally validate the PROSS results (Table 1). A human hERα-LBD variant, covering residues 304–548 of the wild-type sequence and termed ER_WT*_ from here on, was produced as a reference. In this variant, three cysteine residues are replaced by serines (C381S, C417S and C530S) in order to preclude undesired cysteine oxidation and erroneous disulfide bridge formation (Table 1). Variant ER_PRS*_ copies the design of ER_WT*_ and at the same time displays all 24 AA substitutions suggested by PROSS. Two additional variants, i.e. ER_PRS*_(+) and ER_PRS*_(−), were produced to facilitate protein crystallization and structural studies. These variants are identical to ER_PRS*_, but contain one or two additional AA exchanges that have been shown to improve the crystallization behavior of hERα when crystallized with small molecule agonists (in case of ER_PRS*_(+)) or antagonists (ER_PRS*_(−)) (Table 1)^4,32^. Whereas the Y537S substitution present in ER_PRS*_(+) helps to fix helix H12 in the coregulator-binding-active conformation, the substitutions L372R and L536S in ER_PRS*_(−) favor an alternative positioning of H12 as observed in the inactive conformation of hERα (Supplementary Fig. S1).

**Table 1:** hERα-LBD variants used in this study.

hERα variant	Substitutions precluding cysteine oxidation^a^	Substitutions stabilizing distinct conformational states^b^		Substitutions suggested by PROSS^c^
		Agonist-bound	Antagonist-bound	
ER_WT*_	C381S, C417S, C530S			
ER_PRS*_	C381S, C417S, C530S			M315I, V316I, D321E, T334S, S341Y, R363K, T371S, E397D, N407D, N413E, S433E, M437E, N439K, G442R, S450A, E471N, D473E, H474K, V478M, T485A, H488W, L489Y, A493S, T496S
ER_PRS*_(+)	C381S, C417S, C530S	Y537S		
ER_PRS*_(−)	C381S, C417S, C530S		L372R L536S	

^a^AA substitutions with respect to UNIPROT entry P03372-1^48^.

^b^As suggested by Bruning et al.^4^ and Nettles et al.^32^.

^c^Campeotto et al.^25^.

All ER_PRS*_ variants yielded protein amounts in the range of 30–60 mg of pure protein per liter of bacterial cell culture. By contrast, purification of ER_WT*_ resulted in only approximately 10 mg protein per liter (data not shown). Interestingly, and similar to the wild-type protein, all ER_PRS*_ variant proteins co-sedimented with the insoluble cell debris and consequently had to be solubilized with urea prior to any further purification steps. Overall, the purification protocol of all variants closely resembles that of the wild-type hERα-LBD protein^33^.

**Figure 2:**
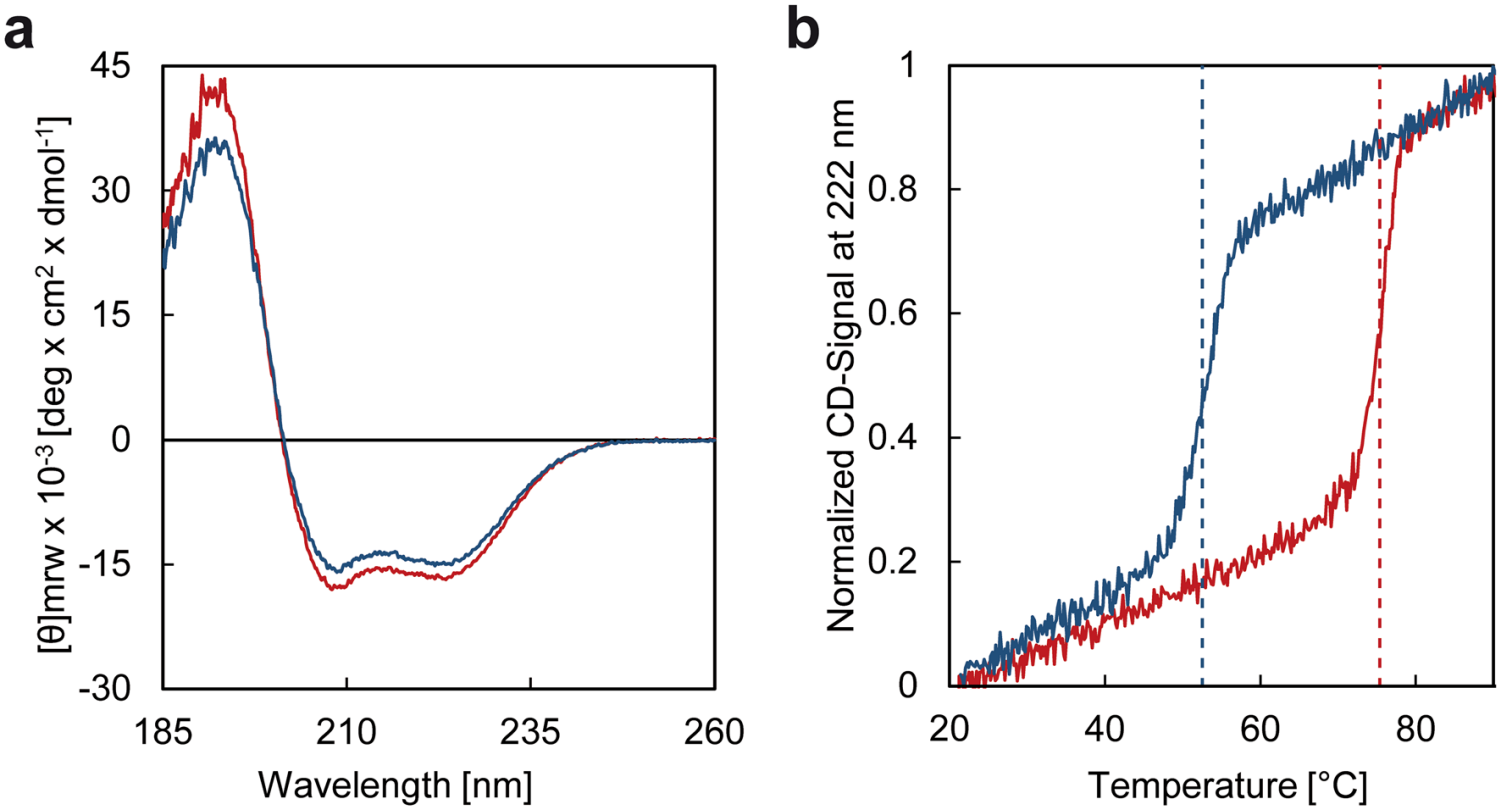
CD characterization of the ER_PRS*_ variant (red) in comparison to the wild-type protein ER_WT*_ (blue). (**a**) Comparison of the molar ellipticity in the range of 185–260 nm of ER_PRS*_ and ER_WT*_. (**b**) Temperature-induced unfolding of ER_PRS*_ and ER_WT*_ as monitored by the normalized CD signal at 222 nm over the temperature range of 20–90 °C. The T_M_ values are indicated by dotted lines. All measurements were performed in triplicate (see also Supplementary Fig. S3).

Circular dichroism (CD) measurements were performed to investigate whether the variant ER_PRS*_ is properly folded. The CD spectra of the wild-type protein ER_WT*_ and of the PROSS-designed variant ER_PRS*_ share the same x axis intercept (201 nm) and show identical curve progressions in agreement with CD spectra of predominantly α-helical proteins (Fig. 2a)^34^. Thus, ER_WT*_ and ER_PRS*_ display highly similar secondary structure compositions and likely the same protein fold (see also below).

To further validate the success of the PROSS design, the thermal stability was monitored by examining the ratio of folded *versus* unfolded protein in a temperature interval of 20–90 °C using identical heating rates, buffer conditions and protein concentrations (Fig. 2b, Supplementary Fig. S3). Whereas wild-type ER_WT*_ unfolds at 52.5 °C, the melting temperature (T_M_) of ER_PRS*_ is considerably higher, namely 75.3 °C. It should be noted that the thermal unfolding of both ER_WT*_ and ER_PRS*_ is not reversible. Therefore, these experiments do not allow discussion of equilibrium thermodynamic stabilities. Nevertheless, these experiments clearly reveal that protein production yields are significantly increased in case of ER_PRS*_ and that the thermal stability of PROSS-designed ER_PRS*_ is about ~ 23 °C higher than that of ER_WT*_.

### Functional in vitro characterisation of ER_PRS*_

**Figure 3:**
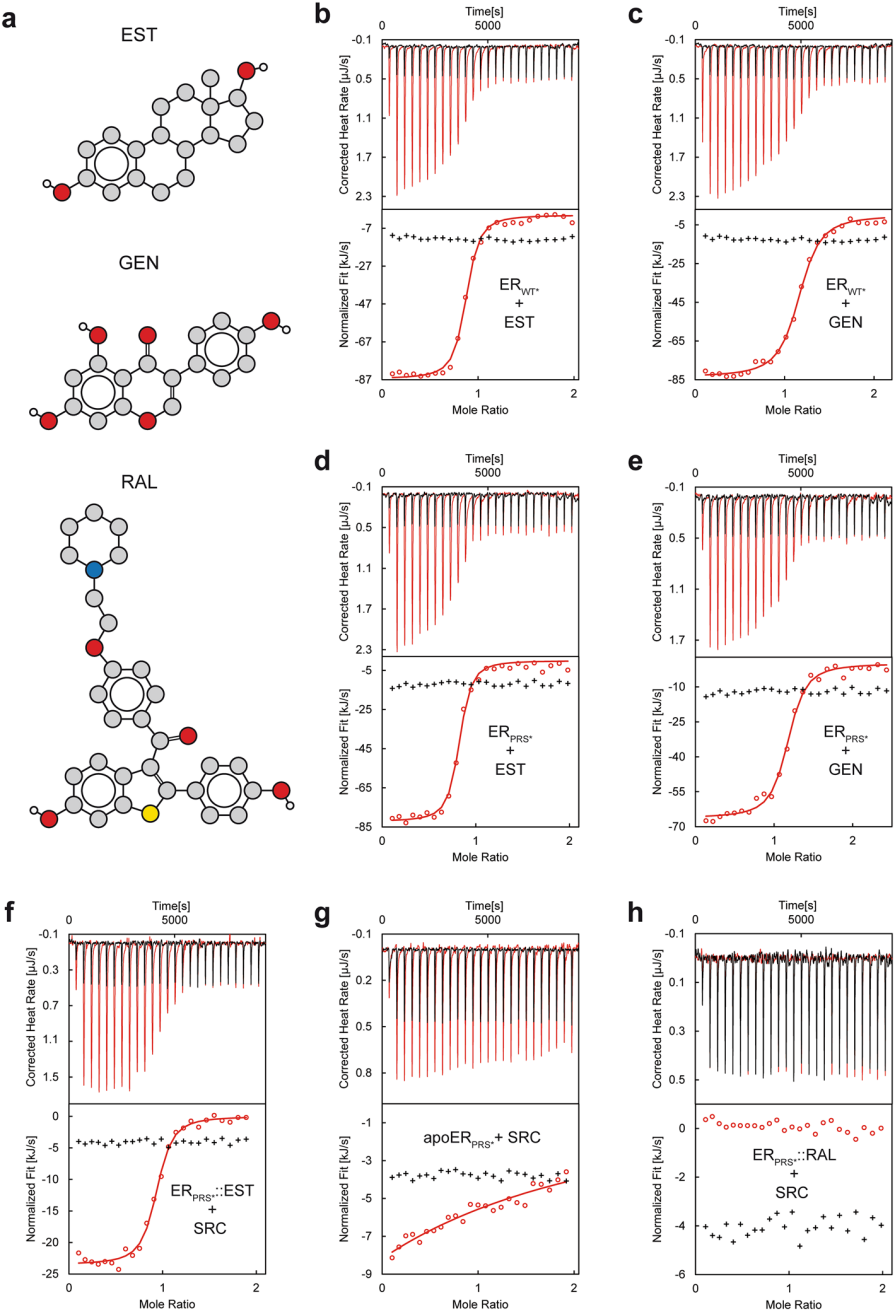
Corroboration of the native-like function of ER_PRS*_ by comparing the affinities of ER_PRS*_ for estradiol and genistein to those of the wild-type protein ER_WT*_ and by analyzing the coactivator affinity modulation of ER_PRS*_ by hERα effectors. (**a**) Structure sketches of the native agonist estradiol (EST), the phytoestrogen genistein (GEN) and the antagonist raloxifene (RAL). ITC measurements of ER_WT*_ titrated into estradiol (**b**) and genistein (**c**) and ER_PRS*_ into estradiol (**d**) and genistein (**e**). ITC traces obtained upon titration of ER_PRS*_ incubated with estradiol (**f)**, raloxifene (**h**) and apo ER_PRS*_ (**g**) into the hERα coactivator peptide SRC-2. The ligand titrations, the integrated heats (circles) and the fitted binding models (solid lines) are highlighted in red. The corresponding blank titrations and integrated heats (crosses) are colored in black. The molar ratios were calculated with respect to the concentrations of monomeric ER_PRS*_ and ER_WT*_.

Detailed affinity measurements were conducted in order to investigate whether the ligand and protein interaction profile of hERα-LBD is retained in ER_PRS*_ in spite of the presence of 24 AA substitutions. In case of the ligand genistein, only a small difference in binding affinities is observed between ER_WT*_ and ER_PRS*_ (K_d_ of 160 nM versus 143 nM) (Table 2, Fig. 3). Notwithstanding this, the thermodynamic parameters ΔH and TΔS differ considerably between the two proteins, with higher absolute values observed for ER_WT*_ (ΔH = − 83.0 kJ/mol, TΔS = − 44.2 kJ/mol) than for ER_PRS*_ (ΔH = − 66.3 kJ/mol, TΔS = − 27.2 kJ/mol). In case of the natural ligand estradiol, both proteins share nearly identical affinities (79 nM and 84 nM for ER_WT*_ and ER_PRS*_, respectively) (Table 2). The thermodynamic parameters ΔH and TΔS show again a similar trend as previously observed for genistein. However, in case of estradiol, the differences in ΔH and TΔS appear only marginal and amount to about 5 kJ/mol in both the enthalpy and entropy term (Table 2).

**Table 2:** Agonist and antagonist-binding parameters and modulation of coactivator SRC-2 binding in ER_WT*_ and ER_PRS*_.

Protein	Incubated ligand		Titrated ligand	K_d_ [nm]	n	ΔH [KJ/mol]	− TΔS [J/mol/K]^a^
ER_WT*_	Apo	–	Estradiol	79	0.7	− 86.1	45.5
ER_PRS*_	Apo	–	Estradiol	84	0.7	− 81.1	40.7
ER_WT*_	Apo	–	Genistein	160	1.1	− 83.0	44.2
ER_PRS*_	Apo	–	Genistein	143	1.2	− 66.3	27.2
ER_PRS*_	Apo	–	SRC-2	> 100,000	1^b^	–	–
ER_PRS*_	Estradiol	Agonist	SRC-2	401^c^	0.9	− 24.1	− 12.5
ER_PRS*_	Raloxifene	Antagonist	SRC-2	–^d^	–	–	–

^a^T = 298.15 K.

^b^Stoichiometry fixed to 1.

^c^This compares well to the value of 175 nM reported by Bramlett et al. for wild-type hERα^36^.

^d^No detectable interaction.

The function of hERα-LBD extends beyond that of a mere ligand-binding protein since ligand binding triggers in addition an allosteric rearrangement of H12 that either favors or disfavors coregulator binding (Supplementary Fig. S1). In order to investigate whether this allosteric mechanism is retained in ER_PRS*_, additional affinity measurements were performed with ER_PRS*_ and a coactivator peptide corresponding to residues 686–699 of the SRC-2 protein and containing the sequence of SRC-2’s nuclear receptor interaction motif 2^3^. SRC-2-binding affinities were measured for ER_PRS*_ alone, ER_PRS*_ incubated with the agonist estradiol and incubated with the antagonist raloxifene (Table 2). In its apo form, ER_PRS*_ binds to SRC-2 but with an affinity that can be estimated to be lower than 100 µM. Due to this low affinity, the Wiseman c-value was < 0.5 in the experimental setup, and therefore the data allowed only for an estimation of the dissociation constant^35^. This weak interaction can be completely abrogated by adding the antagonist raloxifene to the system. By contrast, for ER_PRS*_ bound to the agonist estradiol, the affinity increases to 401 nM (Fig. 3, Table 2). The latter value compares well to the previously reported value of 175 nM^36^. In view of this pronounced ligand-triggered modulation of coactivator binding, it seems reasonable to conclude that the allosteric signal conduction is not influenced by the mutations and that variant ER_PRS*_ appears fully functional.

### Structural chracterisation of ER_PRS*_

**Figure 4:**
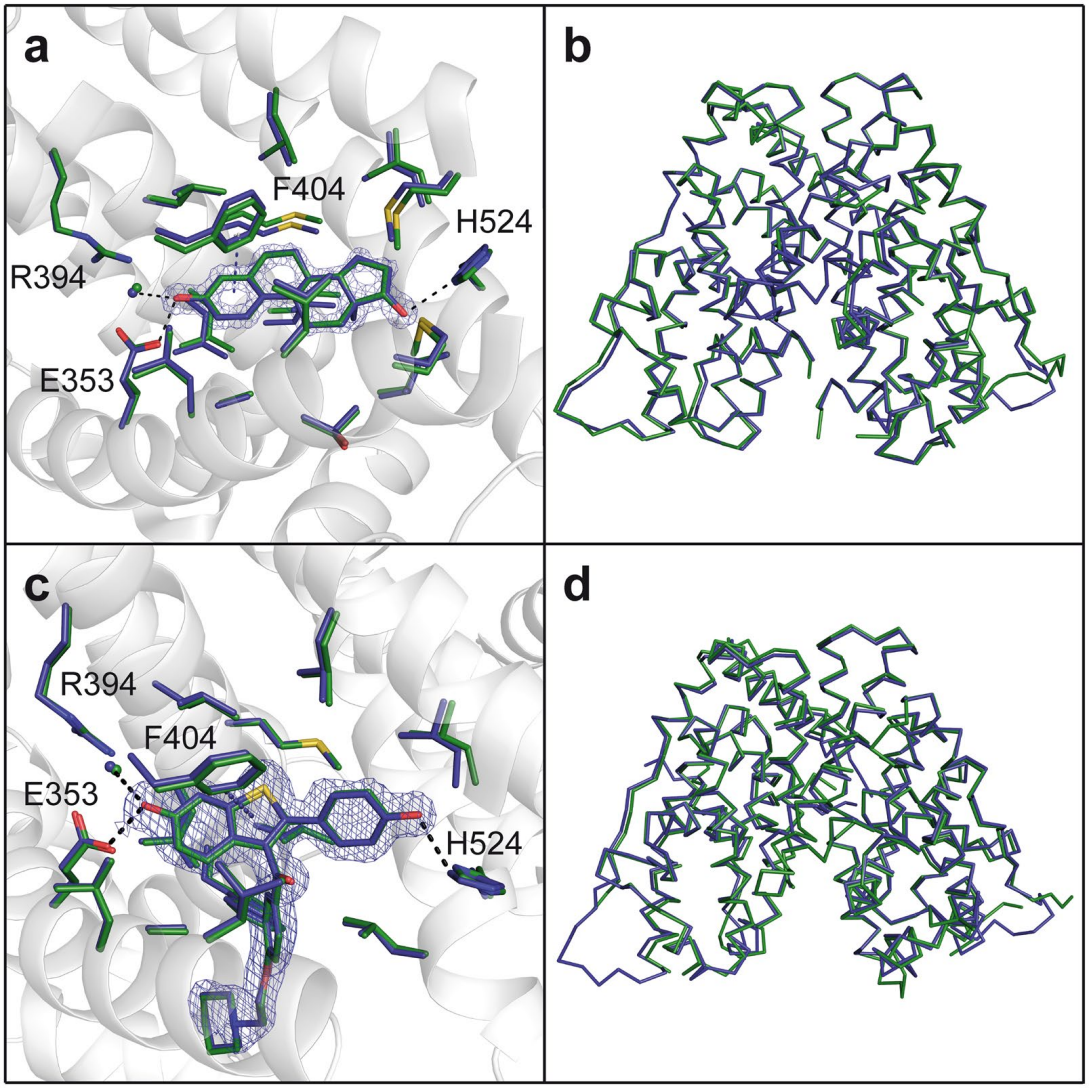
Structural comparison of ER_PRS*_ and hERα-LBD. Detailed comparison of the estradiol (**a**) and raloxifen (**c**)-bound ligand-binding sites of ER_PRS*_ with the wild-type hERα structure (PDB entries 3UUD and 2QXS, respectively)^4,37,47^. All residues involved in ligand binding are represented as green sticks for hERα and as blue sticks for ER_PRS*_. Water molecules interacting with the ligands are shown as spheres and selected hydrogen bonds are displayed as black lines. The electron density (2 F_obs_–F_clac_) of the ligands is depicted at 2.5 σ for estradiol and 1.0 σ for raloxifen and is displayed within 1.6 Å of any ligand atom. The overall structure comparison shows the Cα ribbon superimposition of hERα (green) and ER_PRS*_ (blue) in complex with estradiol (**b**) and raloxifene (**d**).

The ER_PRS*_ variants ER_PRS*_(+)_,_ and ER_PRS*_(−) were crystallized in order to visualize the structural implications of the PROSS-suggested substitutions. As stated before, the conformation of the LBD is stabilized in either the canonical active (ER_PRS*_(+)) or inactive (ER_PRS*_(−)) conformation in these two variants, thereby considerably improving their crystallization behavior^4,32^. Structures of ER_PRS*_(+) were determined in complex with the coactivator peptide SRC-2 and two different agonist ligands, namely either in presence of the ligand estradiol or the phytohormone genistein, and refined to resolutions of 1.45 and 1.33 Å, respectively. The structure of ER_PRS*_(−) was solved in complex with the antagonist raloxifene at a resolution of 1.6 Å (Table 3). Homomeric dimers are observed in all crystal structures, and each structure is nearly undistinguishable from the wild-type hERα-LBD structures in complex with the identical ligands and coactivator peptide available from the protein databank (PDB) (Fig. 4, Supplementary Fig. S4, Supplementary Table S2)^37^. No pronounced changes can be detected in the overall structures of these 12 helices-containing proteins (H1–H12) as shown by the low RMSD_Cα_ values of 0.5–0.8 Å obtained upon superposition of all equivalent Cα atoms in the compared structures (Supplementary Table S2). This also extends to the position and conformation of the SRC-2 peptide in the estradiol and genistein complexes. A few minor conformational deviations can be observed in some surface loops in the various structures (Fig. 4, Supplementary Fig. S4).

**Table 3:** Crystallographic data collection and refinement statistics.

Structure	ER_PRS*_(−)::RAL	ER_PRS*_(+)::EST::SRC	ER_PRS*_(+)::GEN::SRC
**Data collection**			
Beamline	BESSY II 14.1	BESSY II 14.2	BESSY II 14.2
Wavelength [Å]	0.9184	0.9184	0.9184
Space group	P 1	P 12_1_1	P 12_1_1
Unit cell [Å, °]	a = 48.6 b = 51.8 c = 57.5 α = 97.8 β = 113.5 γ = 110.4	a = 55.8 b = 82.8 c = 58.7 α = γ = 90 β = 108.8	a = 55.9 b = 81.5 c = 58.5 α = γ = 90 β = 108.5
Resolution range [Å]	42.80–1.60 (1.66–1.60)*	26.41–1.45 (1.50–1.45)	27.73–1.33 (1.38–1.33)
Unique reflections	56,359 (5535)	88,797 (8813)	112,549 (11,151)
Multiplicity	3.7 (3.6)	11.3 (11.6)	6.8 (6.8)
Completeness [%]	93.6 (92.1)	99.4 (99.0)	98.9 (98.5)
R-meas [%]	6.1 (149.9)	9.9 (342.7)	6.3 (226.9)
R-pim [%]	3.1 (76.6)	2.9 (99.2)	2.4 (86.3)
<I/σI>	11.81 (0.86)	12.45 (0.83)	15.87 (0.84)
CC1/2	0.999 (0.478)	0.999 (0.353)	1 (0.360)
CC*	1 (0.804)	1 (0.722)	1 (0.728)
Wilson B-factor [Å^2^]	25.9	21.7	18.3
**Refinement**			
R_work_/R_free_ [%]	18.5/19.5	14.9/18.6	14.2/17.8
CC (work)	0.967 (0.705)	0.976 (0.705)	0.975 (0.699)
CC (free)	0.967 (0.589)	0.958 (0.595)	0.975 (0.643)
*No. of atoms (non-H)*			
Macromolecules	3863	4225	4255
Ligands	103	131	119
Solvent	289	429	435
*RMSD from ideal geometry*			
Bonds [Å]	0.003	0.006	0.005
Angles [°]	0.52	0.76	0.77
*Ramachandran statistics*			
Favored [%]	98.9	99.8	99.6
Outliers [%]	0.0	0.0	0.0
Clashscore	3.7	2.8	2.7
Average B [Å^2^]	42.1	33.6	26.8
Ligands	50.1	45.1	31.7
No. of TLS groups	17	–**	–**

*Statistics for the highest-resolution shell are shown in parentheses.

**Refinement of individual anisotropic B-factors for all atoms excluding hydrogens.

As expected from the closely matching ligand-binding affinities of ER_PRS*_ and ER_WT*_, the fine details of all ligand-binding interactions are retained between variants ER_PRS*_ and wild-type hERα-LBD. The superposition of the different binding sites shows that the positioning of the ligands and the surrounding AAs are perfectly congruent between ER_PRS*_ and wild-type hERα-LBD (Fig. 4, Supplementary Fig. S4). Not only are all specific polar contacts between the ligands and the AAs Arg394, Glu353 and His524 conserved but also the T-shaped π-stacking between the aromatic portions of the different ligands and the Phe404 benzene ring. Moreover, water molecules bridging between ligands and protein side chains appear also fully conserved.

ER_PRS*_ displays 24 substitutions and these substitutions increase the thermal stability of hERα-LBD by ~ 23 °C in comparison to ER_WT*_. The crystal structures show that 20 of the 24 substituted AAs are surface-located, and the mutated AAs introduce four additional surface charges and the formation of five novel salt bridges. Between two and four substitutions appear to either improve the packing or the extent of the hydrophobic core. Without doubts, additional mutational experiments will be required to identify the exact contributions of newly introduced interactions to the increased thermal stability. Nevertheless, a number of structural features appear worthwhile highlighting.

**Figure 5:**
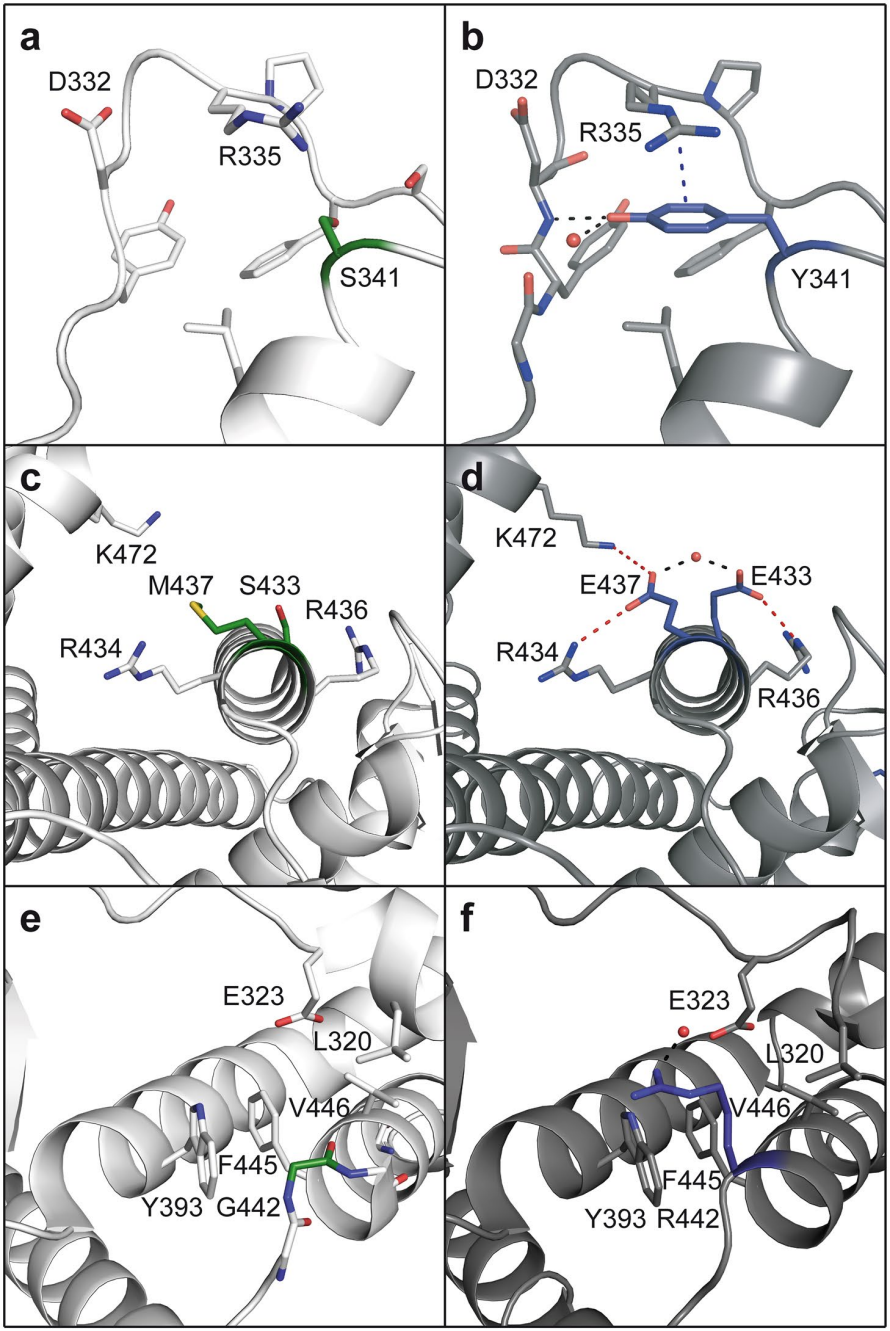
Structural implications of selected PROSS substitutions. hERα (PDB entry 2QA8)^32,37^ is shown on the left side (light gray) and the corresponding region of ER_PRS*_ on the right (as observed in the structure of ER_PRS*_(+) in complex with genistein, dark gray). Salt bridges are colored red, hydrogen bonds black and π-stacking interactions blue. The S341Y substitution is shown in (**a**) and (**b**), the S433E and M437E substitutions in (**c**) and (**d**) and G442R in (**e**) and (**f**). The mutated AAs are highlighted in dark blue and the corresponding AAs of the wild-type in green.

The S341Y substitution at the beginning of helix H3 introduces a feature that closely resembles the tyrosine corner observed in β-sandwich structures such as for example in FNIII domains (Fig. 5a,b)^38,39^. In ER_PRS*_, the hydroxyl group of Tyr341 forms a hydrogen bond with the main chain nitrogen of Asp332 from the preceding loop. At the same time, the benzene ring of Tyr341 is within the right distance to Arg335 to form an inter-side chain cation-π interaction and thereby possibly stabilizing the positioning of Tyr341 and in turn the loop that interconnects H2 to H3 (Fig. 5a,b). Conversely, Ser341 is not able to form a similar interaction in wild-type hERα-LBD.

The substitutions S433E and M437E allow for the formation of a novel network of salt bridges not present in wild-type hERα-LBD (Fig. 5c,d). While the salt bridges involving Arg436 and Arg434 are formed with residues that are all displayed from the same helix H8, an additional inter-subunit salt bridge with a distance slightly over 4 Å is formed between Glu437 and Lys472’ from the second monomer, and the latter interaction might therefore contribute to the stabilization of the dimer assembly (Fig. 5c,d).

Finally, the substitution G442R located in the N-terminal turn of helix H8 introduces an additional surface charge and a water mediated interaction with Glu323 in ER_PRS*_ (Fig. 5e,f). At a first glance, this substitution appears unlikely since this exchange introduces a dramatic change in size, charge and polarity. Moreover, a glycine residue can explore a wider range of main chain dihedral angles than non-glycine residues. However, inspection of Gly442 in wild-type hERα-LBD reveals that Gly442 displays α-helical dihedral angles and these remain unaltered upon exchange of this residue against arginine in ER_PRS*_ (data not shown). The hydrophobic portion of the side chain of Arg442 in ER_PRS*_ forms a number of additional hydrophobic interactions with residues such as Leu320, Trp393, Phe445 and Val446, which cannot be formed when a glycine is present at position 442 (Fig. 5e,f).

Of the ER_PRS*_ AAs discussed above, Tyr341 displayed the lowest relative frequency in the phylogenetic analysis (0.3%) while relatively low values were also observed for Glu433 (7.4%) and Arg442 (5.0%) (Supplementary Fig. S2). However, the structures reveal clear benefits arising from these substitutions, in testimony of the importance of the atomistic side chain-packing calculations included in the PROSS algorithm^18^.

## Discussion

The PROSS server calculations proved to be highly beneficial for the stabilization of the hERα-LBD. Using PROSS, a protein variant ER_PRS*_ was designed that displays multiple enhanced general characteristics. ER_PRS*_ can be produced with high yields in *E. coli* and displays a drastically improved thermal stability. Furthermore, ER_PRS*_ and more precisely ER_PRS*_(+) together with agonists and coactivator peptide and ER_PRS*_(−) in complex with an antagonist crystallize readily and yielded crystals diffracting reproducibly to resolutions of up to 1.33 Å. Notably, in case of ER_PRS*_(+), crystals grew within hours. This significantly improved protein handling and crystallization behavior shows promise for the integration of such variants into semi-automated experimental flow schemes aiming at identifying novel estrogen receptor modulators. Such flow schemes could also target the identification of potent estrogen receptor degraders^40^. The latter structurally destabilize wild-type hERα and trigger the degradation of hERα in the cell. Here, our ER_PRS*_ variants might be beneficial due to their enhanced stability. Compared to hERα, the PROSS-designed variant ER_PRS*_ also seems to be better suited for in vitro characterizations such as high-throughput binding assays due to the high stability, production yields and the substitution of surface cysteines, abrogating the need for the addition of reducing agents, which can significantly impact the experimental results. Since hERα is involved in many pathological processes such as cancer and osteoporosis, the aggregated improved characteristics of ER_PRS*_ show promise for facilitating the further exploration of hERα as a drug target.

Despite many published examples of proteins stabilized by tools such as PROSS or Fireprot^41^, no such study has been published to the best of our knowledge on a protein with such a complex allosteric regulatory mechanism as present in hERα. Moreover, with about 10% of all AAs mutated, it was highly questionable whether the conformational flexibility required for the allosteric regulation of hERα function could be preserved in ER_PRS*_. In the present study, it is shown that ER_PRS*_ retains all functional and structural features characterizing the wild-type protein. The affinity and thermodynamic characteristics of the interaction between ER_PRS*_ and its native agonist estrogen as well as to the phytoestrogen genistein remain unaltered by the 24 substitutions. This also extends to the structural binding characteristics of the antagonist raloxifene and to the resulting inhibition of coactivator binding.

In addition to small molecule ligand binding, hERα functions as a ligand-triggered protein–protein interaction switch. To check whether the allosteric coupling between coregulator protein binding and small molecule effector binding is preserved in ER_PRS*_, the SRC-2 coactivator peptide-binding affinity was investigated in the presence of an agonist, an antagonist and in the absence of any affinity-modulating small molecule. Agonist-bound ER_PRS*_ displays a coactivator-binding affinity of 401 nM, whereas the affinity is in the low mM range in the absence of any small molecule effector (> 0.1 mM). Moreover, no detectable coactivator-binding affinity is observed upon binding of the antagonist raloxifene. This clearly shows that the small molecule-triggered modulation of the binding affinity of hERα to its coactivator peptide is perfectly retained in ER_PRS*_.

The crystal structures clearly demonstrate that the ligand-triggered switching between the active and inactive conformation of hERα is fully preserved in ER_PRS*_. This is underlined by the low RMSD_Cα_ values between the structures of ER_PRS*_ and hERα bound to the corresponding ligands. This appears remarkable since the hERα-LBD was optimized using solely the agonist-bound structure for the PROSS calculations, namely hERα in complex with estradiol and SRC-1. At the same time, the antagonist-bound structure differs significantly from the agonist-bound structure due to the distinct repositioning of H12, which is essential for hERα function. The preserved repositioning of H12 might be a direct consequence of the inclusion of phylogenetic considerations in the PROSS calculations. These render it unlikely that highly conserved residues important for the intramolecular signal transduction and conformational changes are being substituted. These anticipated beneficial effects resulting from the inclusion of phylogenetic data beg the question of whether phylogeny should be used in a broader manner and more readily during the design of binding pockets and the optimization of catalytic sites.

The advances achieved by applying PROSS to hERα might be readily transferable to other NR-LBDs since NRs share extended sequence and structure similarities. The very high number of available NR sequences allows for extended and detailed phylogenetic analyses and it appears likely that these significantly contributed to the success of PROSS in the redesign of hERα. One could argue that, by using a PSSM matrix for defining the set of AAs to be considered at individual positions, the wealth of possibilities offered by all twenty natural AAs is unnecessarily restricted. However, in the case of hERα, the PROSS approach still allowed for various unexpected substitutions and structural features, as highlighted by the posterior phylogenetic analysis and the crystal structures. It is possible that the tremendous increase in thermal stability of ~ 23 °C is caused by a combined effect of the five newly introduced salt bridges, the newly introduced tyrosine corner and the four additional surface charges. As previously observed, all these structural features can have a significant impact on protein stability^42,43^. However, it has to be mentioned that salt bridges can also decrease protein stability^44^. Possibly, the phylogenetic analysis included in PROSS helped to prevent the introduction of detrimental point mutations (see above).

ER_PRS*_ described here reemphasizes the potential of PROSS for the design of more stable protein variants. Extending beyond previous successes, the design and characterization of ER_PRS*_ impressively shows that the phylogeny-based approach of PROSS can be also successfully applied to the optimization of allosterically regulated proteins, even though our understanding of intramolecular allosteric communication pathways still remains fragmental and the nature of allostery remains controversially discussed to the present day^45,46^. Given the importance of NRs in cell homeostasis and signal transduction, it can be expected that the success reported here will encourage and facilitate further exploration of these key proteins as drug targets.

## Methods

### Bioinformatical engineering of ER_PRS*_

The PROSS server was used with default settings and the structure of hER-LBD in complex with its natural ligand estradiol and bound to the coactivator peptide SRC-1 (PDB code: 3UUD) as an input model^37,47^. AA substitutions within a 5 Å distance of the dimerization interface or within a 8 Å radius of either the bound ligand or residues interacting with the coactivator peptide were excluded from the calculations in order to preclude adverse effects on protein function.

### Protein production and purification

The partially optimized protein production and purification protocol parallels that published by Ferrero et al*.*^33^. The codon-optimized genes of the wild-type hER-LBD (residues 304–548, UNIPROT entry P03372-1) or of the different variants (Table 1) were inserted into the multiple cloning site of a pET15b vector^48^. In all plasmid constructs, a N-terminal hexahistidine tag and a segment encoding for a tobacco etch virus (TEV) protease cleavage site precede the segment encoding for the target protein.

The plasmids harboring the different variants were transformed into chemically competent *E. coli* BL21 (DE3) Star cells (Invitrogen, Carlsbad, USA). Terrific Broth cultures were inoculated with overnight precultures and were grown at 37 °C prior to the induction of protein expression at an OD_600_ of 1.5 with 0.5 mM IPTG and continuing shaking for 20 h at 18 °C. The cells were harvested by centrifugation and resuspended in 50 mM HEPES, 500 mM NaCl, 20 mM imidazole, 1 mM EDTA, 0.5 mM AEBSF, pH 8.0. The cells were disrupted by sonication, and the solution centrifuged at 8000×*g* for 1 h. The supernatant was discarded, and the pellet was resolubilized in 100 mM NDSB 201, 50 mM HEPES, 50 mM NaCl, 20 mM imidazole, 4 M urea, 1 mM EDTA, 0.5 mM AEBSF and pH 8.0 and centrifuged at 100,000×*g* for 1 h.

The supernatant was loaded onto a preequilibrated HisTrap FF column (GE Healthcare, Boston, USA), and the column washed with 50 mM HEPES, 500 mM NaCl, 20 mM imidazole and pH 8.0. The protein variants were eluted using a step gradient ranging from the washing buffer to 50 mM HEPES, 300 mM NaCl, 500 mM imidazole, pH 8.0. The peak fractions were pooled. The hexahistidine tag was removed by adding TEV protease to the protein solution at a mass ratio of 1:1,000 while dialyzing the protein solution against 50 mM HEPES, 500 mM NaCl, 20 mM imidazole, 2.5 mM DTT, 0.5 mM EDTA, pH 8.0 for 16 h and subsequently against 50 mM HEPES, 500 mM NaCl, 20 mM imidazole, pH 8.0 for 4 h. To remove the hexahistidine-tagged TEV protease and any remaining uncleaved protein, a second affinity chromatography step was performed analogously to the first one, but pooling the flow-through fraction instead. As a final purification step, a size exclusion chromatography was performed with a HiLoad 26/600 Superdex 75 pg column (GE Healthcare) using a 25 mM HEPES, 150 mM NaCl, pH 8.0 buffer. The pure protein fractions were pooled, flash-frozen in liquid nitrogen and stored at − 80 °C.

### Circular dichroism

The secondary structure content and the thermal stability of the wild-type protein and the stabilized mutant were investigated using a J-815 CD spectrometer (JASCO, Pfungstadt, Germany). Prior to the experiments, the protein solutions were incubated with dextran-coated charcoal (Sigma-Aldrich) while agitating for at least 6 h, followed by a buffer exchange into a 10 mM KH_2_PO_4_/K_2_HPO_4_, pH 8.0 buffer using a PD MiniTrap G-25 column (GE Healthcare). CD spectra for the secondary structure determination were recorded by accumulating 10 ellipticity measurements of a 5 µM protein solution between 185 and 260 nm with 1 mm optical path length and 20 nm/min scanning speed.

The denaturation experiments were performed in triplicate with a protein concentration of 0.75 µM and 10 mm path length. The samples were heated at a speed of 1 degree per minute in the temperature interval of 20–90 °C, and the ellipticity was monitored at 222 nm. The melting temperatures were determined using the software Spectra Manager (JASCO).

### Isothermal titration calorimetry

Isothermal titration calorimetry (ITC) experiments were performed with a Standard Volume Nano ITC (TA Instruments, New Castle, USA) and a 24 K gold cell. The protein solutions were incubated first with dextran-coated charcoal at 16 °C for 24 h while gently agitating in order to remove any lipophilic contaminant potentially occupying the effector-binding site. After centrifugation, the solutions were dialyzed repeatedly against 100 mM KH_2_PO_4_/K_2_HPO_4_, 150 mM NaCl, pH 7.2.

To determine the thermodynamic parameters of the interaction between the protein variant and the ligands estradiol and genistein, the ligands were dissolved in the dialysis buffer of the corresponding protein sample, and the ligand solutions were heated to 80 °C while agitating for 1 h. The ligand concentrations were determined photometrically, and the protein solution was titrated subsequently into the ligand solution.

The affinity between the protein variant and the coactivator peptide SRC-2 was investigated in the presence of the agonist estradiol, the antagonist raloxifene and in the absence of any effector. The coactivator peptide with the sequence KHKILHRLLQDSSS corresponding to residues 686–699 of the SRC-2 protein (UNIPROT entry Q15596) was N-terminally acetylated and C-terminally amidated^3,48^. The peptide was synthesized using Fmoc-based solid-phase synthesis, as previously described^49^. For the measurements in presence of effectors, the protein variant was incubated first with either solid powder of estradiol or raloxifene for 16 h at 16 °C while gently agitating. The protein solution was titrated into the peptide solution in all experiments.

All measurements were performed in triplicate with degassed solutions. Each measurement consisted of 25 incremental titrations (1 × 5 µL, 24 × 10 µL) interspaced by 360 s time intervals at 25 °C and 150 rpm stirring rate. Additionally, blank titrations with protein only were performed and the ITC measurements were corrected using the determined constant. The data were processed using the NanoAnalyze Software v3.11.0 (TA Instruments) with fixed integration intervals and manually checked baselines.

### Crystallization and crystal structure determinations

All protein solutions were incubated first with dextran-coated charcoal, gently rocked for 24 h at 16 °C and subsequently centrifuged. To determine the crystal structures of the stabilized protein in the agonist-bound active conformation, a solution consisting of 350 µM ER_PRS*_(+) and 1.4 mM SRC-2 was prepared in a 25 mM HEPES, 10% glycerol, pH 8 buffer. Either solid genistein or estradiol was added, and the solution incubated for 16 h while agitating. For the structure of the protein stabilized in the antagonist-bound inactive conformation, 700 µM ER_PRS*_(−) in 25 mM HEPES and pH 8 were incubated with solid raloxifene for 72 h while agitating. Screening for crystallization conditions was performed in 96-well plates with commercially available screens using the sitting-drop vapor diffusion technique. Initial hits were optimized manually using the hanging-drop method.

In case of both agonist-bound complexes, single plate-shaped crystals could be obtained within 16 h with droplets consisting of 2 µL protein solution, 2 µL reservoir solution (200 mM NaCl, 100 mM Tris pH 8.5 and 25% polyethylene glycol 3,350) and 0.4 µL water equilibrated over 700 µL reservoir solution. Trapezoid like crystals of ER_PRS*_(−) in complex with raloxifene grew after around 3 months in droplets consisting of 0.2 µL protein solution and 0.4 µL reservoir solution (0.2 M sodium chloride, 0.1 M BIS–TRIS pH 5.5, 25% w/v polyethylene glycol 3,350) equilibrated over 70 µL reservoir solution. All crystals were cryo-protected with 20–30% ethylene glycol and flash-frozen in liquid nitrogen prior to data collection.

Diffraction data sets were collected at the synchrotron beamlines BL 14.1 and BL 14.2 at BESSY-II in Berlin^50^. The raw diffraction images were processed with the program XDS^51^, and the phase problem was solved using the program PHASER within the PHENIX software suite^52^ with previously published structures of wild-type hERα-LBD in complex with estradiol (PDB code: 3UUD) and raloxifene (PDB code: 2QXS) as search models. The models were refined via alternating cycles of automated coordinate refinement with PHENIX and manual building in the program COOT^53^. The RMSD_Cα_ values between the wild-type and the stabilized structures were calculated with LSQKAB from the CCP4 program suite^54^. All structure illustrations were drawn using Pymol^55^.

## Supplementary Information

Supplementary Information.

## Data Availability

Accession code Protein Data Bank: the coordinates and structure factors have been deposited with the Protein Data Bank under accession codes 7NFB, 7NEL, 7NDO.
