# Supplementary material for: A PROSS-designed extensively mutated estrogen receptor α variant displays enhanced thermal stability while retaining native allosteric regulation and structure
Source: Sci Rep. 2021 May 18;11:10509. doi: 10.1038/s41598-021-89785-1 (PMC8131754; doi:10.1038/s41598-021-89785-1)
Supplement: Supplementary file 1 — Supplementary Information. [file 41598_2021_89785_MOESM1_ESM.pdf]

Supplementary information file

A PROSS-designed extensively mutated estrogen receptor α variant displays enhanced thermal stability while retaining native allosteric regulation and structure

Mark Kriegel, Hanna J. Wiederanders, Sewar Alkhashrom,
Jutta Eichler and Yves A. Muller

Table S1. Protein and ligand concentrations used to determine the agonist and antagonist-binding parameters and modulation of coactivator SRC-2 binding by ITC.

Titrating solution: Protein variant preincubated with or without a specified ligand*			Molecule present in the titration cell	
Protein variant	Preincubated with ligand**	$c_{protein}$ [µM]***	Ligand	c_{ligand} [µM]
ER_{WT^*}	- none-	100	estradiol	12
		100		13
		100		14
ER_{PRS^*}	- none -	107	estradiol	12
		107		15
		107		13
ER_{WT^*}	- none -	100	genistein	11
		100		13
		100		13
ER_{PRS^*}	- none -	107	genistein	12
		107		16
		107		12
ER_{PRS^*}	- none -	300	SRC-2	44
		300		46
		300		42
ER_{PRS^*}	estradiol	289	SRC-2	41
		289		43
		260		43
ER_{PRS^*}	raloxifene	289	SRC-2	41
		260		43
		258		40

* All experiments were performed in triplicates.
** For the concentration of the preincubated ligand, please see the Methods section.
*** All protein concentrations were calculated using the molecular weight of the monomeric protein.

Table S2. $RMSD_{C\alpha}$ values from the comparison of ER_{PRS*} and hERα-LBD crystal structures.

	hERα-LBD::EST::SRC*	hERα-LBD::GEN::SRC**	hERα-LBD::RAL***
ER_{PRS*}(+)::EST::SRC	0.62 Å****		
ER_{PRS*}(+)::GEN::SRC		0.52 Å	
ER_{PRS*}(-)::RAL			0.83 Å

* Protein databank (PDB) entry code 3UUD[1,2]. In 3UUD and 2QA8 (see below), hERα-LBD displays the same Y537S substitution as present in ER_{PRS*}(+). This substitution stabilizes the active agonist-bound conformation of hERα-LBD[3].
** PDB code 2QA8[3].
*** PDB code 2QXS[4]. In 2QXS, hERα carries the substitution L536S that stabilizes the inactive antagonist-bound conformation of hERα-LBD[4].
**** The $RMSD_{C\alpha}$ values were calculated using the Cα-atom positions of matching residue pairs in the compared structures.

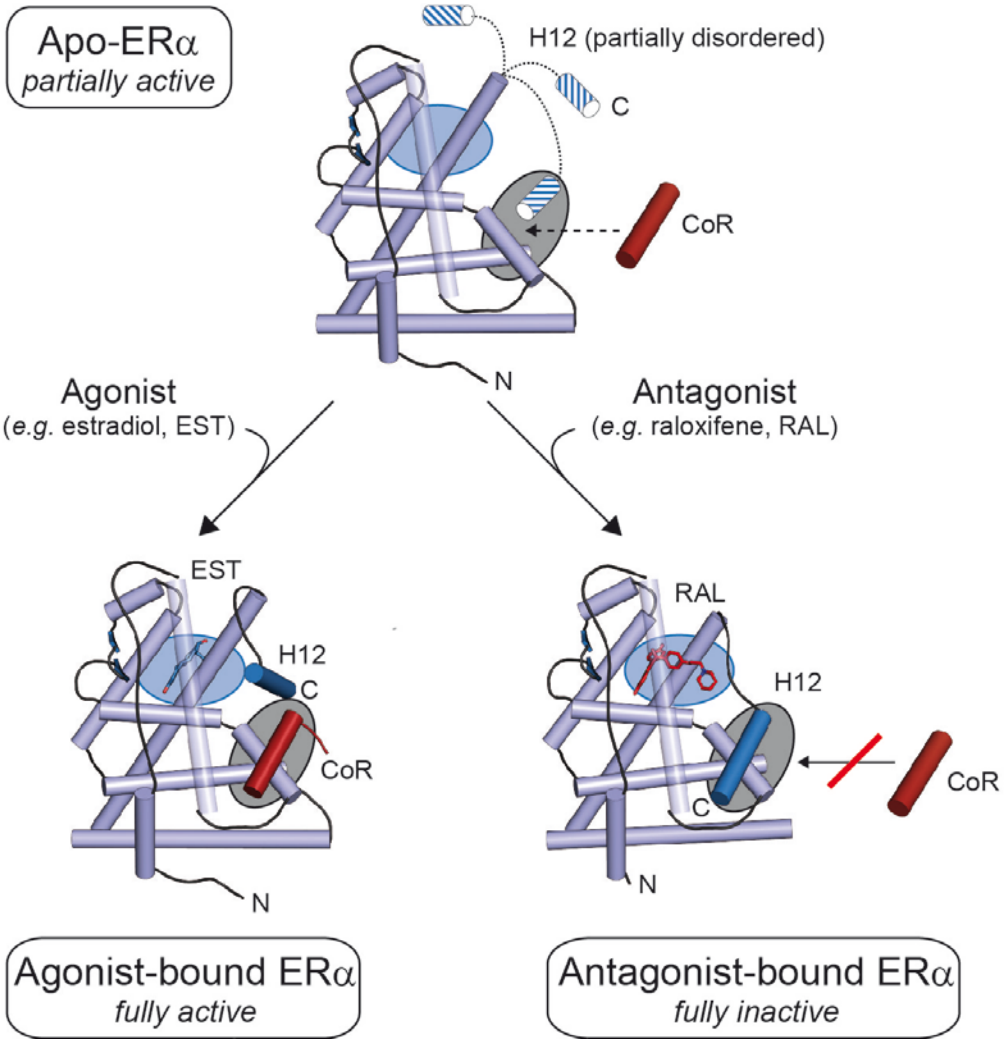

Figure S1. The effect of agonists and antagonists on the coregulator-binding activity of nuclear receptors, in general, and estrogen receptor, in particular. While agonist binding (i.e. estradiol binding) allows the concomitant binding of helix 12 (H12) and of a coregulator protein (CoR) such as the steroid receptor coactivator-2 (SRC-2) protein to the ligand-binding domain (LBD) of the nuclear receptor (active conformation), binding of antagonists such as raloxifene shifts the binding position of H12 to the coregulator protein-binding site thereby inhibiting the binding of SRC-2 (inactive conformation).[4,5]

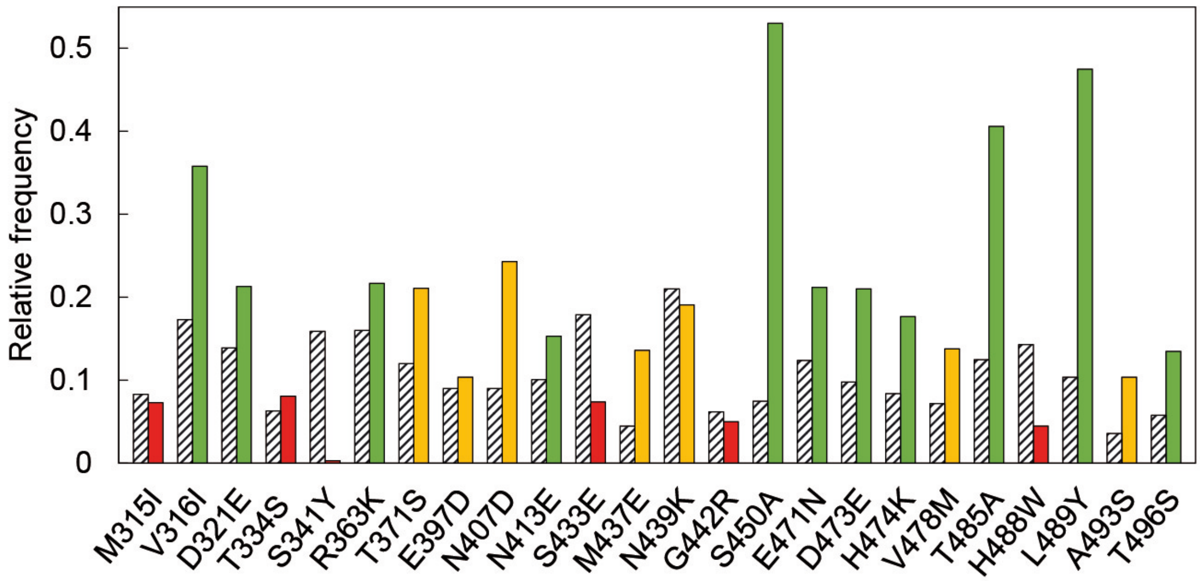

Figure S2. Phylogenetic analysis of the substitutions suggested by PROSS. The relative amino acid (AA) frequency of the native AA (shaded bars) and the frequency of the AA suggested by the algorithm (solid colored bars) are compared at the corresponding positions. The suggested AAs are highlighted in green if they correspond to the most abundant AA observed at this position in a multiple sequence alignment, in yellow if they correspond to either the second or third most frequent AA and else in red. The relative frequencies were derived from a multiple sequence alignment of 422 nuclear receptor sequences.

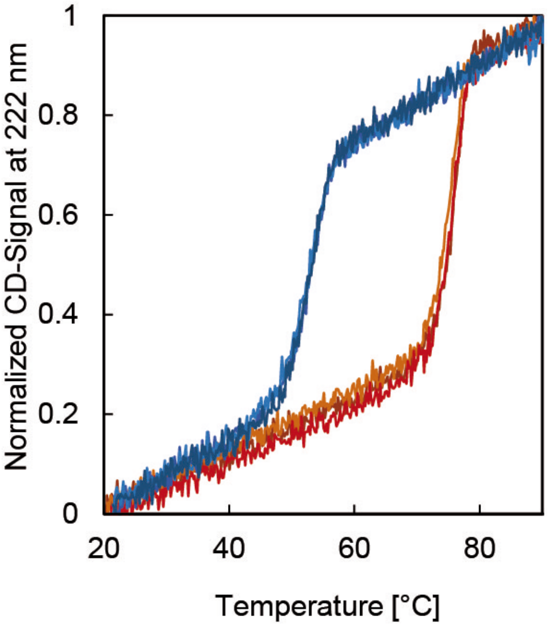

Figure S3. Circular dichroism (CD) comparison between the wild-type protein ER_{WT*} (blue) and the ER_{PRS*} variant (red) in triplicates. Normalized CD signal recorded at 222 nm for ER_{WT*} (blue tones) and ER_{PRS*} (red tones) is shown over the temperature interval between 20 and 90 °C.

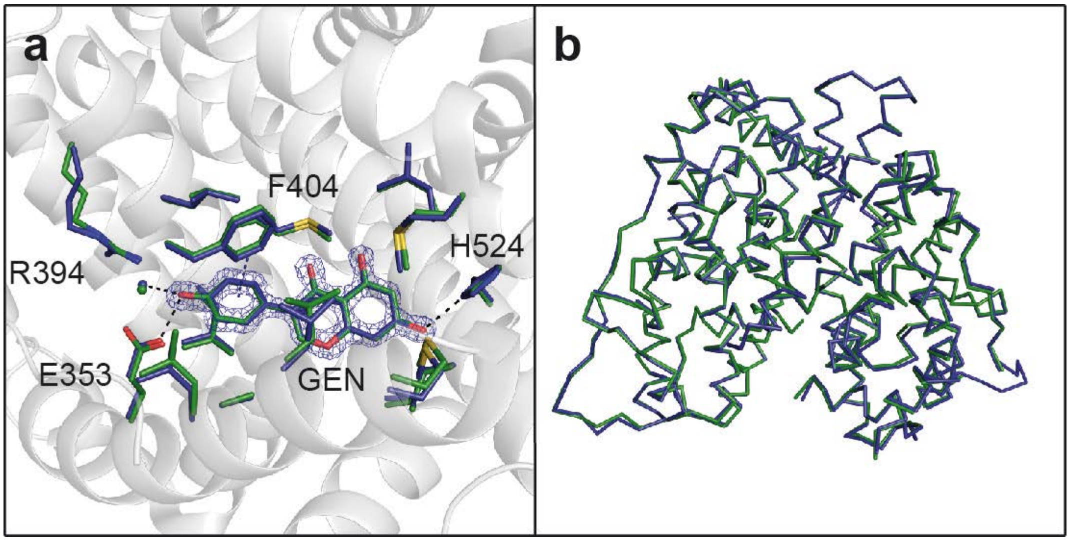

Figure S4. Structural comparison of ER_{PRS*}(+) and hERα-LBD in complex with genistein. (**a**) Detailed comparison of the ligand-binding site of ER_{PRS*}(+) with the wild-type hERα structure (PDB entry 2QA8)[1,3]. All residues involved in ligand binding are represented as green sticks for hERα and as blue sticks for ER_{PRS*}. Water molecules interacting with the ligand are shown as spheres and hydrogen bonds are displayed as black dotted lines. The electron density of genistein (2 F_{obs}- F_{clac}) is depicted at 2.5 σ and is displayed within 1.6 Å of any ligand atom. (**b**) The overall structure comparison shows the Cα ribbon superposition of hERα (green) and ER_{PRS*}(+) (blue).

Supplementary references

1. Rose, P. W. *et al.* The RCSB protein data bank: integrative view of protein, gene and 3D structural information. *Nucleic Acids Res* **45**, D271-D281, doi:10.1093/nar/gkw1000 (2017).
2. Delfosse, V. *et al.* Structural and mechanistic insights into bisphenols action provide guidelines for risk assessment and discovery of bisphenol A substitutes. *Proc Natl Acad Sci U S A* **109**, 14930-14935, doi:10.1073/pnas.1203574109 (2012).
3. Nettles, K. W. *et al.* NFkappaB selectivity of estrogen receptor ligands revealed by comparative crystallographic analyses. *Nat Chem Biol* **4**, 241-247, doi:10.1038/nchembio.76 (2008).
4. Bruning, J. B. *et al.* Coupling of receptor conformation and ligand orientation determine graded activity. *Nat Chem Biol* **6**, 837-843, doi:10.1038/nchembio.451 (2010).
5. Nagy, L. & Schwabe, J. W. Mechanism of the nuclear receptor molecular switch. *Trends Biochem Sci* **29**, 317-324, doi:10.1016/j.tibs.2004.04.006 (2004).
